# Consensus report from the 10th global forum for liver magnetic resonance imaging: multidisciplinary team discussion

**DOI:** 10.1007/s00330-023-09919-z

**Published:** 2023-07-13

**Authors:** Bachir Taouli, Ahmed Ba-Ssalamah, Julius Chapiro, Jagpreet Chhatwal, Kathryn Fowler, Tae Wook Kang, Gesine Knobloch, Dow-Mu Koh, Masatoshi Kudo, Jeong Min Lee, Takamichi Murakami, David J. Pinato, Kristina I. Ringe, Bin Song, Parissa Tabrizian, Jin Wang, Jeong Hee Yoon, Mengsu Zeng, Jian Zhou, Valérie Vilgrain

**Affiliations:** 1 Department of Diagnostic, Molecular, and Interventional Radiology, BioMedical Engineering and Imaging Institute, Icahn School of Medicine at Mount Sinai, New York, NY USA; 2 Department of Biomedical Imaging and Image-Guided Therapy, Medical University of Vienna, Vienna, Austria; 3 Department of Radiology and Biomedical Imaging, Yale School of Medicine, New Haven, CT USA; 4 Department of Radiology, Institute for Technology Assessment, Massachusetts General Hospital, Harvard Medical School, Boston, MA USA; 5 Department of Radiology, University of California San Diego, La Jolla, CA USA; 6 Department of Radiology and Center for Imaging Science, Samsung Medical Center, Sungkyunkwan University School of Medicine, Seoul, South Korea; 7 Global Medical and Clinical Affairs and Digital Development, Radiology, Bayer Pharmaceuticals, Berlin, Germany; 8 Department of Diagnostic Radiology, Royal Marsden Hospital, Sutton, UK; 9 Department of Gastroenterology and Hepatology, Kindai University Faculty of Medicine, Osaka, Japan; 10 Department of Radiology, Seoul National University Hospital and Seoul National University College of Medicine, Seoul, South Korea; 11 Department of Radiology, Kobe University Graduate School of Medicine, Kobe, Japan; 12 Department of Surgery & Cancer, Imperial College London, Hammersmith Hospital, London, UK; Division of Oncology, Department of Translational Medicine, University of Piemonte Orientale, Novara, Italy; 13 Department of Diagnostic and Interventional Radiology, Hannover Medical School, Hannover, Germany; 14 Department of Radiology, West China Hospital, Sichuan University, Chengdu, People’s Republic of China; 15 Recanati/Miller Transplantation Institute, Icahn School of Medicine at Mount Sinai, New York, NY USA; 16 Department of Radiology, Third Affiliated Hospital of Sun Yat-Sen University, Guangzhou; Liver Disease Hospital, Sun Yat-Sen University, Guangzhou, People’s Republic of China; 17 Department of Radiology, Zhongshan Hospital, Fudan University, Shanghai, People’s Republic of China; 18 Liver Cancer Institute, Zhongshan Hospital, Fudan University, Shanghai, People’s Republic of China; 19 Université Paris Cité and Department of Radiology, Assistance-Publique Hôpitaux de Paris, APHP Nord, Hôpital Beaujon, Clichy, France

**Keywords:** Gadoxetic acid, Hepatocellular carcinoma, Magnetic resonance imaging

## Abstract

**Abstract:**

The 10th Global Forum for Liver Magnetic Resonance Imaging was held in October 2021. The themes of the presentations and discussions at this Forum are described in detail in the review by Taouli et al (2023). The focus of this second manuscript developed from the Forum is on multidisciplinary tumor board perspectives in hepatocellular carcinoma (HCC) management: how to approach early-, mid-, and late-stage management from the perspectives of a liver surgeon, an interventional radiologist, and an oncologist. The manuscript also includes a panel discussion by multidisciplinary experts on three selected cases that explore challenging aspects of HCC management.

**Clinical relevance statement:**

This review highlights the importance of a multidisciplinary team approach in liver cancer patients and includes the perspectives of a liver surgeon, an interventional radiologist, and an oncologist, including illustrative case studies.

**Key Points:**

*• A liver surgeon, interventional radiologist, and oncologist presented their perspectives on the treatment of early-, mid-, and late-stage HCC.*

*• Different perspectives on HCC management between specialties emphasize the importance of multidisciplinary tumor boards.*

*• A multidisciplinary faculty discussed challenging aspects of HCC management, as highlighted by three case studies.*

## Multidisciplinary tumor board perspectives: How I approach HCC treatment (early-, mid-, and late-stage HCC)

### Perspectives from a liver surgeon

The goals in HCC management from the surgeon’s perspective are to: refine candidate selection for resection and transplantation; convert cases to resection or transplantation by downstaging/downsizing; assess biologic markers as surrogates of tumor behavior; and, ultimately, decrease recurrence rates and improve survival.

### Candidate selection

The Barcelona Clinic Liver Cancer (BCLC) staging system is the mainstay for candidate selection for HCC treatment [1, 2]. The BCLC system classifies patients with HCC into five stages: very early, early, intermediate, advanced, and terminal; this system uses established prognostic variables, including tumor status, liver function, and performance status [1, 2].

Patients with very early (BCLC 0, single nodule ≤ 2 cm, Child-Pugh A) or early-stage HCC (BCLC A, single nodule any size or ≤ 3 nodules ≤ 3 cm, Child-Pugh A–B) are treated with local curative treatments: resection, ablation, or liver transplantation [2, 3].

### Resection

Resection is theoretically an imperfect treatment, given that most patients have diminished liver reserves secondary to hepatitis, and the intrahepatic metastasis rate is high. Appropriate candidates for resection are those patients with Child-Pugh A liver function, absence of portal hypertension (assessed by platelet count and lack of portosystemic collaterals on cross-sectional imaging), and low hepatic venous pressure gradient (< 10 mm Hg) [2]. Generally, patients with multifocal tumors or macrovascular invasion are not ideal candidates because of the low likelihood of cure [4].

Treatment of recurrence remains challenging. Tabrizian et al [5] reviewed survival following recurrence post resection in 661 patients with single nodules, absent extrahepatic spread, and Child-Pugh A liver function at baseline. HCC recurred in 356 (54%) patients, at a median of 22 months. Median 5-year survival in the entire study population was 45%, and HCC recurrence resulted in a 24% decrease in 5-year survival. To assess factors influencing survival following resection, Roayaie et al [6] recorded survival in 9656 patients with newly diagnosed HCC in the BRIDGE study, who were classified into four groups by the American Association for the Study of Liver Diseases (AASLD) [7] / European Association for the Study of the Liver (EASL) [8] criteria: (A) ideal resection candidates who were resected, (B) ideal resection candidates who were not resected, (C) non-ideal resection candidates who were resected, and (D) non-ideal resection candidates who were not resected. Groups B and D were treated by ablation, embolization, locoregional, and systemic treatments. Median 5-year survival in Groups A, B, and C was 65%, 55%, and 35%, respectively. Multivariate analysis of ideal candidates for resection (A + B) revealed a nearly two-fold lower risk of mortality for resection than for other treatments. In patients who underwent resection, severe liver dysfunction (portal hypertension, Child-Pugh B/C class) and advanced tumor characteristics (multiple tumors, macrovascular invasion, extrahepatic spread) were associated with a detrimental effect on survival.

The use of neoadjuvant therapy, such as transarterial radioembolization (TARE) or immunotherapy, prior to resection is gaining interest [9]. The goals of such studies to date have been to refine candidate selection, potentially convert borderline cases to resection, and ultimately improve survival. Some groups advocate the use of immunotherapy if one of the following surrogates of aggressive tumor biology are met: functional liver reserve (FLR) < 40%, alfa-fetoprotein (AFP) > 400 ng/mL, infiltrative tumor, macrovascular invasion, multifocal disease, or presence of satellite lesions. Marron et al recently reported a phase II trial of neoadjuvant cemiplimab (anti-programmed cell death protein 1 [PD-1]) monotherapy in 21 patients with resectable HCC. Three of 20 patients had a partial response and all others maintained stable disease—supporting the design of larger trials in the future [10].

### Liver transplantation

The Milan criteria for eligibility for transplantation in patients with HCC comprise: (1) a single tumor at least 2 cm and not > 5 cm or (2) up to three tumors, none > 3 cm, in the absence of macrovascular invasion and extrahepatic spread [11]. These criteria have remained the benchmark for candidate selection for more than two decades [11]. Mazzaferro et al reported 5-year survival rates of ~ 70% in patients meeting the Milan criteria [11].

Over time, the Milan criteria have been criticized as being too restrictive. Other morphometric criteria utilizing varying degrees of change in lesion size and number were subsequently reported, although all these lacked markers of tumor biology and were unable to stratify patients accurately [3]. Over time, the focus has shifted to incorporate markers of tumor biology, such as AFP or response to locoregional therapy, into transplant selection guidelines. Sapisochin et al [12], for example, demonstrated that markers of tumor biology (namely, tumor confined to liver, no systemic cancer-related symptoms, no poorly differentiated tumors) as criteria for liver transplantation provided 5-year survival outcomes (68%) similar to the Milan criteria (78%). An AFP level > 500 ng/mL predicted poorer outcomes in both groups. Additional refinements based on AFP may further improve post transplantation outcomes in downstaging groups.

In intermediate-stage HCC (BCLC B, multinodular, Child-Pugh B), chemoembolization and more recently radioembolization remain the standard of care. However, downstaging approaches that combine expanded transplantation criteria with response to locoregional therapy have moved to the front line for selecting transplant candidates whose tumors exceed the Milan criteria. Yao et al [13] assessed the outcomes of downsizing (mainly by transarterial chemoembolization [TACE]) or radiofrequency ablation [RFA]) in 118 patients with HCC outside the Milan criteria (i.e., single lesion ≤ 8 cm, two or three lesions each ≤ 5 cm with the sum of the largest tumor diameters ≤ 8 cm, four or five lesions each ≤ 3 cm with the sum of the largest tumor diameters ≤ 8 cm, no vascular invasion; known as the UCSF criteria). In patients achieving successful downsizing to within the Milan criteria for transplantation, 5-year survival (56.1%) was similar to a control group who initially met Milan criteria for transplantation (63.3%) [13].

Consistent results were reported in a large multicenter study on 2645 patients undergoing transplantation for HCC [14]. Ten-year post transplantation survival and recurrence rates were 52.1% and 20.6%, respectively, in the successfully downstaged group, versus 61.5% and 13.3% in the Milan criteria group, and 43.3% and 41.1% in a beyond Milan criteria but not a downstaged group (*p* < 0.001). Tumor characteristics (> 3 nodules, diameter > 7 cm) and lack of AFP response prior to transplantation were factors independently associated with downstaging failure.

The response to pre-transplant locoregional therapy has been demonstrated to predict recurrence and survival post transplantation [15]. An analysis by the US Multicenter HCC Transplant Consortium showed that patients with a complete pathologic response (i.e., no viable tumor on explant: *n* = 802) had significantly lower 5-year recurrence post transplantation (5.2% vs. 16.4%; *p* < 0.001) and superior overall survival (75% vs. 68%; *p* < 0.001) than patients without a complete response (*n* = 2637).

The waiting time from HCC diagnosis to liver transplantation also influences the outcome. A multi-cohort study by Mehta et al [16] investigated 911 patients with HCC with variable wait times; the practice in all 3 study centers was to use transarterial chemoembolization as first-line locoregional therapy with the intent for continued treatment to achieve complete tumor necrosis prior to liver transplantation. Mehta et al [16] found that the probability of recurrence within 5 years was 14.5% for a wait time < 6 months, 9.8% for 6–18 months, and 19.0% for > 18 months. The poor outcome associated with early transplantation was attributable to the inclusion of patients with aggressive tumors with a high risk of recurrence, while the poor outcome in patients with a prolonged wait time could be explained by a shift to more aggressive behavior over time. Microvascular invasion, elevated AFP, and explant stage beyond Milan criteria were predictors of recurrence. Halazun et al [17] similarly found significantly reduced 5-year survival in patients in regions with short waiting time (median 1.6 months) than long waiting time (median 7.6 months) among 6160 patients in the United Network for Organ Sharing network, at 66% versus 70%, respectively.

There are limited data on surgery in advanced-stage HCC (BCLC C). Small retrospective studies have shown meaningful outcomes in patients with macrovascular invasion who underwent resection or transplantation. For example, the retrospective study by Assalino et al [18] in 30 patients transplanted following a complete radiologic regression of macrovascular invasion using locoregional therapies reported 60% overall survival at 5 years. Median AFP was the only pre-transplant variable that differed significantly between recurrent and non-recurrent patients.

### Conclusions

Selection criteria for liver resection and transplantation continue to evolve, and surrogates of biological behavior should now be included in decision-making. Improvements in the use of bridging and neoadjuvant therapy have extended the limits of what can be achieved, and downstaging has been implemented into treatment algorithms with excellent long-term outcomes. Recurrence, particularly post resection, remains challenging, and the impact of waiting times on outcomes requires further investigation.

## Perspectives from an interventional radiologist

### Interventional radiology techniques in the treatment of HCC

As discussed above, HCC treatment is commonly based on the BCLC staging system, linking liver function, tumor size, and performance status to an evidence-based treatment algorithm [1, 19]. In this algorithm, interventional radiology techniques are recommended primarily in very early-stage (BCLC 0), early-stage (BCLC A), and intermediate-stage (BCLC B) HCC. However, the strict allocation of therapies is often individualized, due to either patient- or tumor-specific factors and the availability of expertise at each institution (Table 1).

**Table 1:** Summary of interventional radiology techniques in HCC

	Thermal ablation	TACE	TARE
BCLC stage recommendation	BCLC stage 0 and A [19]	BCLC Stage B [19]	Role needs to be defined [19–21] Second-line option in selective BCLC A–C • BCLC 0/A: niche for resection candidates with an inadequate volume of future remnant liver (“radiation segmentectomy”) [4] • BCLC B: poor TACE candidates, large tumors, PV invasion [22] • BCLC C: good liver function, liver-confined disease, no systemic therapy feasible Not recommended as first-line therapy in intermediate or advanced stage [19]
Outcomes	Tumors < 3 cm: survival equal to surgery [19] Favorable safety profile [21] No information about risk factors for recurrence (e.g., microvascular invasion)	Median survival 2.5–4 years, if inclusion criteria are followed strictly [23, 24]	No/minimal ischemic effect; may be performed efficiently and safely in patients with portal vein thrombosis [25–27] Compared with sorafenib, superior safety profile, without difference in survival [28]
Technical considerations	RFA and MWA can be considered equal (MWA potentially advantageous in larger tumors/less vulnerable to heat sink effect) Use is dependent on local expertise [4]	cTACE and DEB-TACE considered equal for overall survival DEB-TACE: greater standardization, increased tumoral concentration, lower systemic absorption Superselective Right patient selection (preserved liver function, the extent of tumor burden, etc.) [23]	Very good cooperation between interventional radiology and nuclear medicine is of great significance Two types of microsphere currently available: • Resin microspheres (slow and careful application necessary, embolic potential) • Glass microspheres (higher dose per sphere, faster application possible) [27]

*Abbreviations: BCLC*, Barcelona Clinic Liver Cancer; *cTACE*, conventional transarterial chemoembolization; *DEB-TACE*, drug-eluting bead transarterial chemoembolization; HCC, hepatocellular carcinoma; *MWA*, microwave ablation; *PV*, portal venous; *RFA*, radiofrequency ablation; *TACE*, transarterial chemoembolization; *TARE*, transarterial radioembolization

### Thermal ablation

Ablation in HCC typically refers to thermal ablation. Under image guidance, an applicator is placed directly in the tumor, inducing a temperature change and irreversible cell damage, and resulting in coagulation necrosis that can be visualized on post interventional imaging. Thermal ablation is recommended in very early-stage and early-stage HCC, especially in candidates not fit for surgery [19]. The decision on ablation versus surgery can be challenging and several factors may favor one over the other [19, 21]. From a technical point of view, RFA and microwave ablation (MWA) can be considered equal in terms of overall success [4]. However, MWA is potentially advantageous for larger tumors, as well as for lesions with close proximity to larger vessels [4].

### Transarterial therapies

Transarterial therapies take advantage of the dual blood supply of the liver and the fact that HCC is almost exclusively vascularized by hepatic arterial supply. Combined intra-arterial infusion of chemotherapy and embolizing materials directly into tumor-feeding arteries leads to a high local concentration and prolonged dwelling time of chemotherapeutic agents. Compared with the best supportive care, TACE improves overall survival and is recommended in patients with intermediate-stage HCC [23, 24]. From a technical point of view, conventional TACE by lipiodol or drug-eluting beads may be considered equal in terms of overall survival [23]. However, TACE by drug-eluting beads is characterized by greater standardization of technique, increased concentration in tumors, and lower systemic absorption rate [23].

TARE, also known as selective internal radiotherapy (SIRT), has emerged as an alternative to TACE. TARE utilizes the intra-arterial application of radioactive Y90-loaded microspheres into tumor-feeding arteries. Compared to TACE, TARE has no or minimal ischemic effects. However, the role of TARE in HCC needs to be better defined [20, 21]. It may be a second-line option, for example, in patients with intermediate-stage HCC who are poor TACE candidates and in selected patients with advanced liver-confined disease [4, 19, 22].

### Bridging and downstaging to liver transplantation

Outside the BCLC framework, all interventional radiology techniques may be used for bridging and downstaging to liver transplantation [19]. In this context, TACE is the technique most often applied. When applied successfully for bridging, TACE reduces the tumor recurrence rate after transplantation [29]. Similarly, if performed for downstaging, post-transplant survival of patients is comparable to the survival of patients initially meeting transplantation criteria [13].

### Gadoxetic acid–enhanced MRI in the context of interventional tumor treatment

Patients with known or suspected HCC should be referred to and discussed at a multidisciplinary tumor board (Table 2). Imaging is requested at the initial diagnosis, including contrast-enhanced MRI, preferably with gadoxetic acid at some institutions, because of its superior accuracy for diagnosis and treatment planning, especially in the context of curative treatment (see the SORAMIC trial below [30]). If the MRI findings are unclear, contrast-enhanced CT (CECT) or CEUS may be requested. Depending on the results of tumor staging, patients are then referred for surgical, interventional, oncologic, or radiation treatment.

**Table 2:** Summary of multidisciplinary tumor board perspectives on early-, mid-, and late-stage HCC treatment

BCLC stage	Primary recommendation	Alternative treatment
0, A	Local therapy: resection or ablation	TARE: niche for resection candidates with inadequate future remnant liver
B	Transarterial therapy: TACE or TARE	TARE in poor TACE candidates with large tumors, portal vein invasion
C	Systemic therapy	TARE in selective patients with good liver function, liver-confined disease, no systemic therapy feasible

*Abbreviations: BCLC*, Barcelona Clinic Liver Cancer; *HCC*, hepatocellular carcinoma; *TACE*, transarterial chemoembolization; *TARE*, transarterial radioembolization

In case of curative treatment, imaging follow-up is performed, again by MRI, preferably with gadoxetic acid at some institutions. In the case of palliative treatment, imaging follow-up is performed by CT. Treatment results and imaging results are again discussed at the multidisciplinary tumor board and the treatment strategy may be modified or maintained.

### The SORAMIC trial

The SORAMIC (Sorafenib and Micro-therapy Guided by Primovist Enhanced MRI in Patients With Inoperable Liver Cancer) trial is a prospective, randomized phase II study in patients with HCC, consisting of two therapeutic studies and one diagnostic-imaging study. The recently published diagnostic-imaging study compared the accuracy of gadoxetic acid–enhanced MRI and CECT for treatment decisions in 538 patients with suspected HCC [30]. Patients were stratified to curative (RFA ± sorafenib) or palliative (sorafenib ± TARE) treatment based on the number of lesions and the tumor size at imaging review performed by two reader groups (R1, R2).

The accuracy of treatment decisions was significantly higher with gadoxetic acid–enhanced MRI, at 83.3% (R1) and 81.2% (R2), than with CT, at 73.4% and 70.8%, respectively. Similarly, the number of lesions detected with gadoxetic acid–enhanced MRI was significantly higher than with CT. Image quality was slightly inferior using MRI than CT, although this had no influence on the accuracy of treatment decisions [30]. The authors noted that, for optimal patient assignment to local ablative therapies or resection, a complete assessment of any HCC or borderline nodule is mandatory, and they refer to the Asia-Pacific Association for the Study of the Liver guidelines that recommend gadoxetic acid–enhanced MRI over extracellular contrast media-MRI as a first-line diagnostic test [31].

### Conclusions

HCC treatment should always be in the context of a multidisciplinary approach. Treatment decisions are based on the BCLC staging system but may be tailored to individual patient needs. Interventional radiology techniques are primarily recommended in very early-, early-, and intermediate-stage HCC, but may also be applied outside the BCLC framework for bridging and downstaging to liver transplantation. Gadoxetic acid-enhanced MRI may be beneficial in HCC diagnosis and treatment decision-making, especially in the context of curative treatment.

## Perspectives from an oncologist

### Opportunities and challenges of treatment sequencing

There have been rapid developments in the systemic therapy of HCC in recent years [32]. Systemic therapy should now be the standard of care in patients with advanced/unresectable HCC (BCLC C), preserved liver function (Child-Pugh A), and good performance status (Eastern Cooperative Oncology Group Performance Status [ECOG PS] 0–1) [2, 19].

### Efficacy

The combination of atezolizumab (anti-programmed death ligand 1 [PD-L1] antibody) and bevacizumab (anti-vascular endothelial growth factor [VEGF] antibody) in the IMbrave150 study resulted in a median overall survival of 19.2 months in 336 patients with advanced unresectable HCC [33, 34], and should now be offered to all eligible patients as first-line treatment [19, 35]. The median overall survival of patients treated with sorafenib in the IMbrave150 study was 13.4 months [34]. First-line tyrosine kinase inhibitor (TKI) therapy, such as sorafenib or lenvatinib, should now be reserved for patients who are ineligible for combination immunotherapy (including patients with untreated varices) or who prefer oral therapy [35]. Real-world use of atezolizumab plus bevacizumab has shown reproducible safety and efficacy in advanced HCC [36].

Careful monitoring is required for asymptomatic progression on systemic therapy. There is currently no level I evidence on second-line therapy post immunotherapy, although observational evidence suggests TKI therapy to be an option following unsuccessful checkpoint inhibitor therapy [37]. The American Society of Clinical Oncology (ASCO) and European Society for Medical Oncology (ESMO) guidelines state that, until data are available, second-line therapy with a TKI may be recommended for appropriate candidates [19, 35]. If patients received first-line therapy with sorafenib or lenvatinib, second-line treatment with another TKI such as cabozantinib or regorafenib, or the VEGF receptor 2 inhibitor, ramucirumab, or combined atezolizumab and bevacizumab is recommended [35]. However, many patients are untreatable following TKI therapy because they deteriorate in Child-Pugh score or performance status.

Future directions in the treatment of advanced HCC include potential combinations of TKIs and immune checkpoint inhibitors [38]. Early-phase studies of anti-PD-1 plus TKI combinations, including for instance pembrolizumab and lenvatinib, have led to an appealing 36% overall response rate in patients with advanced disease [39]. However, LEAP-002, the phase III randomized controlled study of pembrolizumab plus lenvatinib, failed to demonstrate a significant advantage in terms of progression-free or overall survival compared to lenvatinib, despite leading to a median overall survival of 21.2 months [40]. Other studies of anti-PD-L1 plus TKI combination such as COSMIC-312, evaluating atezolizumab plus cabozantinib, led to a disappointing median overall survival value of 15.4 months in the combination group versus 15.5 months in the sorafenib arm [41]. To date, the only anti-PD-1 plus TKI combination shown to lead to a significant progression-free and overall survival benefit is the combination of camrelizumab plus rivoceranib, where combination immunotherapy led to a median overall survival of 22.1 months against 15.2 months achieved for the sorafenib control arm, the longest duration ever reported in a first-line trial in HCC [42].

Another potential therapeutic strategy associated with a significant overall survival benefit compared to sorafenib is the combination of the PD-L1 inhibitor durvalumab plus tremelimumab, a CTLA-4 inhibitor [43]. The HIMALAYA study reported the superiority of single-dose priming of tremelimumab followed by maintenance durvalumab treatment over sorafenib, with the combination achieving a median overall survival of 16.4 months versus 13.8 months with sorafenib [43]. Anti-PD-L1 monotherapy with durvalumab was also shown to be non-inferior to sorafenib in HIMALAYA [43], and the results of the non-inferiority study RATIONALE-301 evaluating tislelizumab versus sorafenib confirm this view [44], supporting a potential role for PD-1/PD-L1-targeting agents as an alternative to first-line TKI therapy in patients who are not eligible to combinations. There is no head-to-head comparison of various immunotherapy regimens in advanced HCC; however, network meta-analyses suggest the superiority of combination immunotherapy approaches over PD-1 monotherapy and TKIs, reinforcing the importance of first-line therapy in advanced HCC [45].

We are starting to consider how to integrate these more potent therapies in earlier stages of HCC. The combination of immunotherapy with TACE is supported by the potential for locoregional therapy to increase tumor immunogenicity [46], although there is no level 1 data in this particular setting [47].

### Safety and toxicity

One of the key considerations in careful decision-making is to balance efficacy and toxicity. Toxicity is profoundly different between TKI and checkpoint inhibitor treatments [48, 49]. For TKIs, toxicity can be on-target or off-target; sometimes there is a relationship between toxicity and response, especially with on-target toxicities such as skin and hypertension [48]. Although these toxicities are non-hematologic, so that patients do not become immunosuppressed, they can affect the quality of life, especially in patients exposed to this therapy in the long term [48]. Checkpoint inhibitors often have an immune-mediated mechanism of action in toxicity, which can be multisystem and occur at any dose level [49]. Toxicity can potentially be irreversible and life-threatening, and may also occur after discontinuation [49].

Checkpoint inhibitors (e.g., anti-PD-1 and anti-CTLA-4 antibodies) are associated with synergistic immunotoxicity when used in combination, producing higher proportions of grade 3, 4, and even 5 adverse events compared to their use as monotherapy. Anti-angiogenic combinations of PD-1 and pure VEGF inhibitors are more often associated with additive toxicity. Immuno-TKI combinations (PD-1 plus molecularly targeted agents/multikinase inhibitors) are associated with additive toxicity [50].

There are currently no biomarkers for patient selection [51, 52]. Inevitably the oncologist, hepatologist, and multidisciplinary team must decide which patients will receive TKI or immunotherapy, but predicting toxicity represents a diagnostic and therapeutic challenge in these patients [53].

### Approach to systemic therapy

The approach to systemic therapy should be fundamentally linked to the treatment goals. Can we cure patients and, if not, can we improve their quality of life? Can we prolong life by integrating systemic therapy with other treatment modalities? Patient selection for a particular therapy is strongly based on HCC stage, liver function, and patient factors including, importantly, patient preference, as well as on comorbidities and possible etiology.

### Conclusions

Systemic therapy should be the standard of care in patients with advanced/unresectable HCC, preserved liver function (Child-Pugh A), and good performance status (ECOG PS 0–1). Combined atezolizumab and bevacizumab should be offered to all eligible patients, reserving first-line TKI therapy for patients’ ineligible for combination immunotherapy or who prefer oral therapy. Access to immunotherapy in the second line (PD-1 inhibitors with or without a CTLA-4 inhibitor) is restricted to certain countries and, while not supported by survival benefit, should be discussed on a case-by-case basis, in light of the evidence of disease-modulating activity in advanced HCC. The use of immunotherapy in earlier stages of HCC should be considered in the context of clinical trials [54].

## Multidisciplinary tumor board perspectives: How I approach HCC treatment—case studies

Three selected case studies were discussed by the following experts to explore challenging aspects of HCC management.

- Valérie Vilgrain, radiologist—session chair
- Masatoshi Kudo, hepatologist
- David J. Pinato, oncologist
- Kristina I. Ringe, interventional radiologist
- Parissa Tabrizian, hepato-pancreatic-biliary (HPB)/transplant surgeon
- Bachir Taouli, radiologist
- Jian Zhou, hepatic surgeon

### Case 1: 61-year-old male with genetic hemochromatosis, compensated cirrhosis, and 2.5 cm HCC

#### Professor Vilgrain

**Fig. 1:**
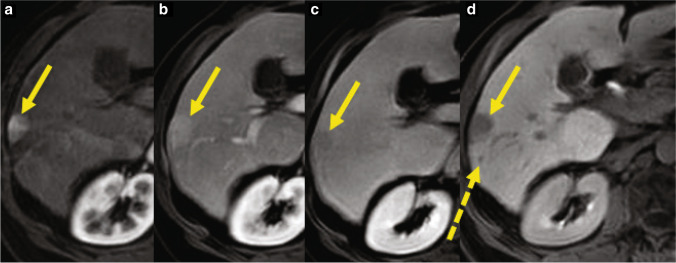
Sixty-one-year-old male with cirrhosis secondary to genetic hemochromatosis and HCC. Gadoxetic acid-enhanced T1-weighted images during the arterial (**a**), portal venous (**b**), transitional (**c**), and hepatobiliary phases (**d**) demonstrate an arterially hyperenhancing lesion (continuous arrows) in the right hepatic lobe, without portal venous washout, with vague hypointensity on the transitional phase, and marked hypointensity on the hepatobiliary phase, compatible with probable HCC, LIRADS 4 according to LiRADS 2018 classification. In addition to the index lesion, there is a punctate hypointense nodule posteriorly on HBP compatible with a satellite lesion (discontinuous arrow on **d**). *Abbreviations: HBP,* hepatobiliary phase; *PVP,* portal venous phase; *TP,* transitional phase

This is a 61-year-old male with a genetic hemochromatosis with compensated cirrhosis, and during the surveillance US, a nodule was detected. Multiphasic gadoxetic acid–enhanced MR showed a lesion compatible with probable HCC, with a satellite lesion (Fig. 1). Here, we discuss the different treatment options.

#### Professor Tabrizian

We know this is a well-compensated cirrhotic patient, assuming no contra-indication to surgery. There is a single 2.5-cm lesion with a satellite lesion, which may increase the risk of recurrence. The options include resection versus ablation; however, my first choice would be surgery, given the presence of a satellite lesion, with the possibility of neoadjuvant immunotherapy or radiation segmentectomy (based on TARE) before surgery.

#### Professor Ringe

I agree, this is definitely a patient for curative treatment. We have a small HCC measuring 2.5 cm and, if we had only that lesion, studies have shown that ablation and surgery have similar outcomes for tumor sizes of up to 3.0 cm. It is important to note that the satellite lesion should be included in the safety margin if interventional therapy is considered. However, I would favor surgery in this case (wedge resection). Ablation could be an alternative if the patient was not resectable due to medical reasons.

#### Professor Vilgrain

If we consider tumor ablation for treatment, would you use a specific technique? Would you pay attention to enlarge the margins regarding the tumor? In other words, would you use some technical refinements or a classical tumor ablation technique?

#### Professor Ringe

From a technical point of view, you could use either RFA or MWA. I personally tend to prefer MWA because we have a more homogeneous zone and it has been shown to be advantageous when you have large vessels next to it. So, with a tangential approach to the lesion, I would ensure that we have enough safety margin. I think that would be the most important factor to consider for ablation in this case.

#### Professor Kudo

I would consider surgery because the lesion is close to the liver surface and there is a tiny satellite lesion. However, there could be more occult lesions not detected by gadoxetic acid–enhanced MRI, for which intraoperative US or CEUS with SonoVue/Sonazoid could be used and thus may orient the surgical resection.

#### Professor Vilgrain

**Fig. 2:**
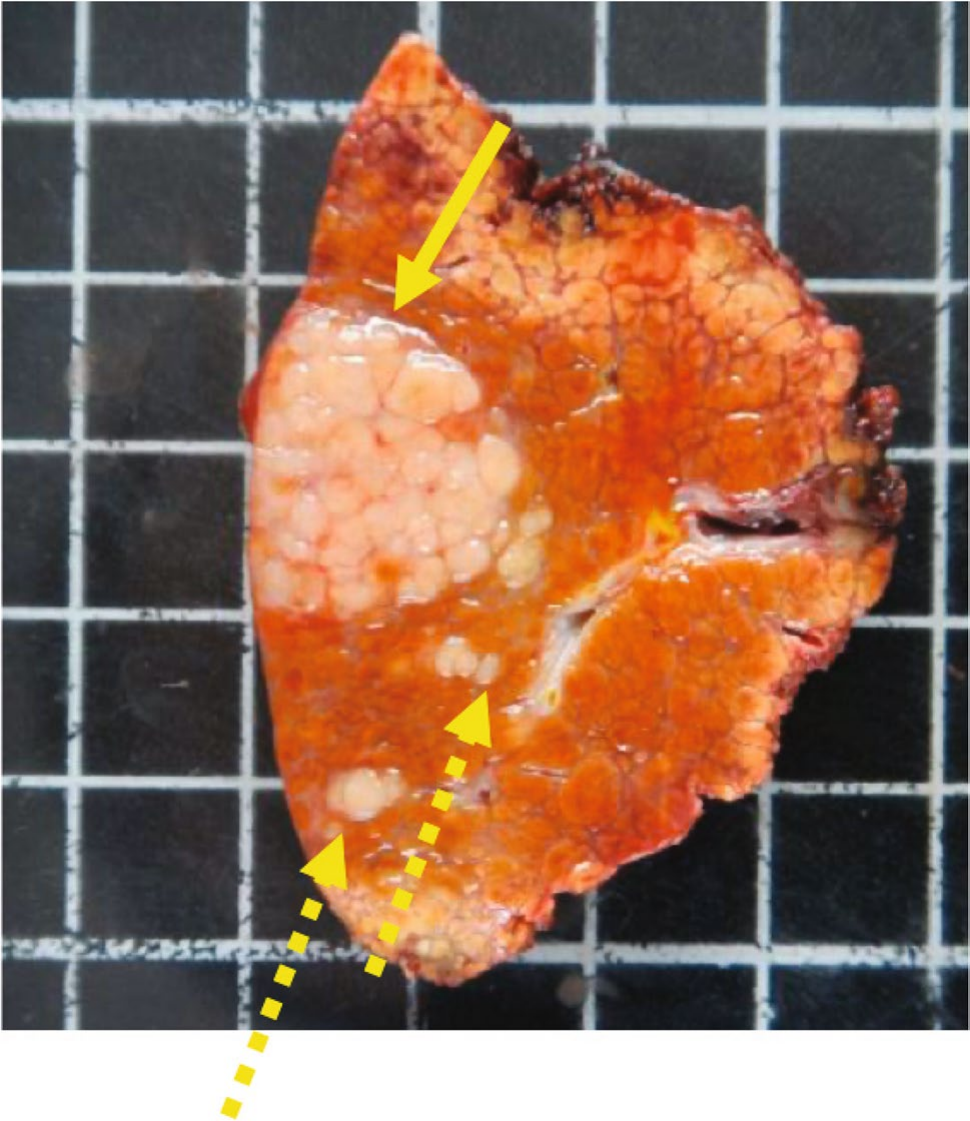
Same patient as in Fig. 1. Gross picture (×20) of right bisegmentectomy specimen demonstrates cirrhotic liver, index HCC (continuous arrow) with several satellite lesions (discontinuous arrow). *Abbreviation: HCC,* hepatocellular carcinoma

The patient underwent anatomic resection that confirmed the diagnosis of HCC. The gross pathologic picture (Fig. 2) shows several small nodules within 1–2 cm from the main tumor, which made resection the best option in this case. Knowing that this is a small HCC but with a high risk of recurrence, would you consider adjuvant treatment?

#### Dr. Pinato

The STORM trial (Sorafenib as Adjuvant Treatment in the Prevention Of Recurrence of Hepatocellular Carcinoma) investigating adjuvant sorafenib after resection or ablation was negative [55], and there are no other data on adjuvant TKI therapy. Recently, there has been a lot of interest in anti-PD-1 monotherapy and potential combinations in other cancers (e.g., renal cell carcinoma or melanoma), in which these therapies are highly effective. However, the jury is still out as to whether this could be an effective adjuvant treatment for patients with HCC. At the moment, the answer would be no.

### Case 2: 60-year-old male with NASH cirrhosis (Child-Pugh B) and two HCC lesions (5.5 cm and 1.6 cm)

#### Professor Vilgrain

**Fig. 3:**
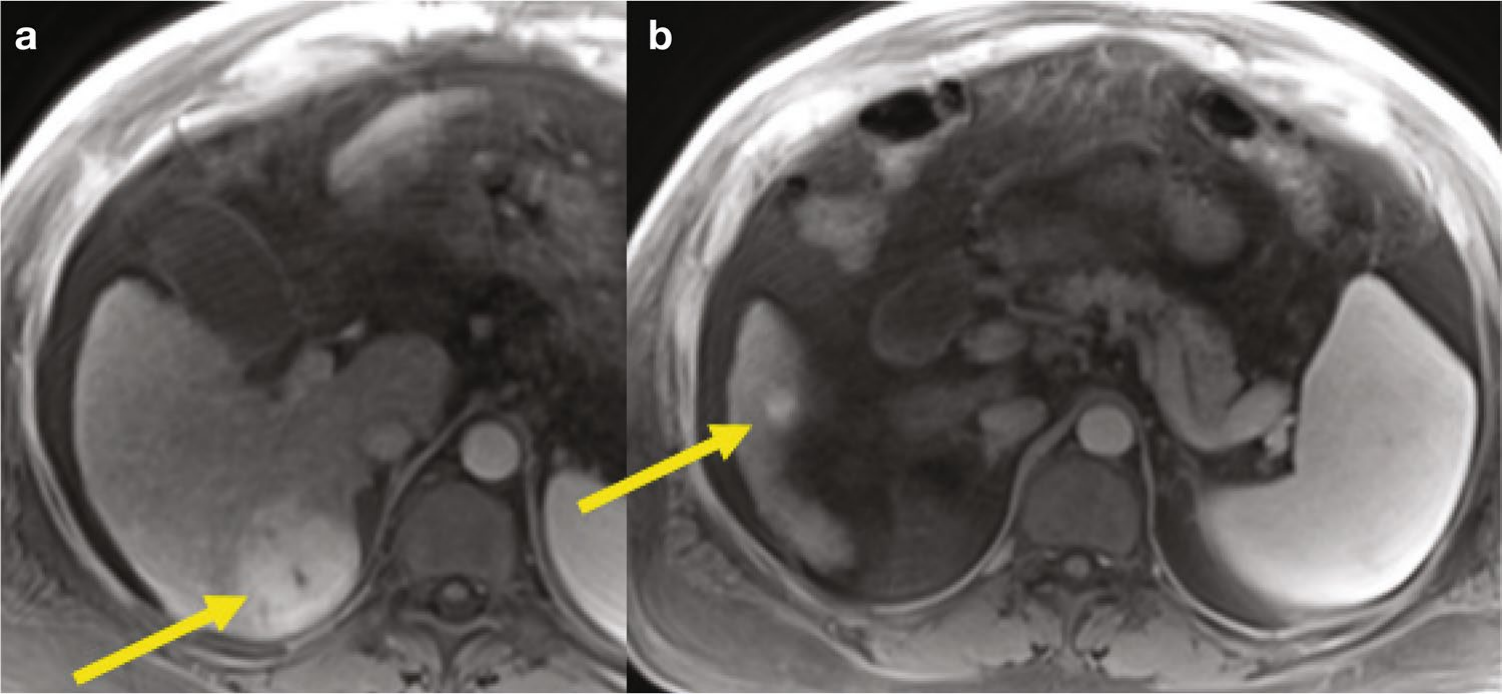
Sixty-year-old male with NASH cirrhosis and HCC. Gadoxetic acid–enhanced T1-weighted images during the arterial phase at two different levels demonstrate two arterially hyperenhancing lesions in the right hepatic lobe (**a**: segment 7 and **b**: segment 6, arrows), compatible with HCC (later phases not shown). There is background cirrhosis and perihepatic ascites. *Abbreviation: HCC,* hepatocellular carcinoma

We have a patient with two HCCs located in the right hepatic lobe (5.5 cm in segment 7 and 1.6 cm in segment 6; Fig. 3). One may consider locoregional therapy to downgrade the patient before transplant. Would you consider TACE or radioembolization (TARE)?

#### Professor Ringe

I agree, this is a good indication for locoregional therapy with the objective of downgrading the tumor to a place where he can be transplanted. The options include TACE and TARE. European guidelines advocate TACE as the primary treatment in patients with intermediate-stage HCC, with TARE as an alternative. TACE in combination with ablation can also be considered.

#### Professor Tabrizian

This patient is not a good candidate for surgical resection. At our institution, we favor TARE over TACE, and we will consider transplantation after successful downstaging.

#### Professor Vilgrain

Based on delegates’ comments, we can see that the opinion between TACE and TARE is split, depending on the institution. Some patients are poor responders to TACE. Do you think this patient will respond to TACE, regarding tumor characteristics, such as size, number of tumors, hypervascularity, or infiltrative pattern?

#### Professor Ringe

It is probably a borderline case. So far, on the images shown, I have not seen macrovascular invasion or an infiltrative pattern. If the tumor was larger, I would have recommended TARE.

#### Professor Vilgrain

**Fig. 4:**
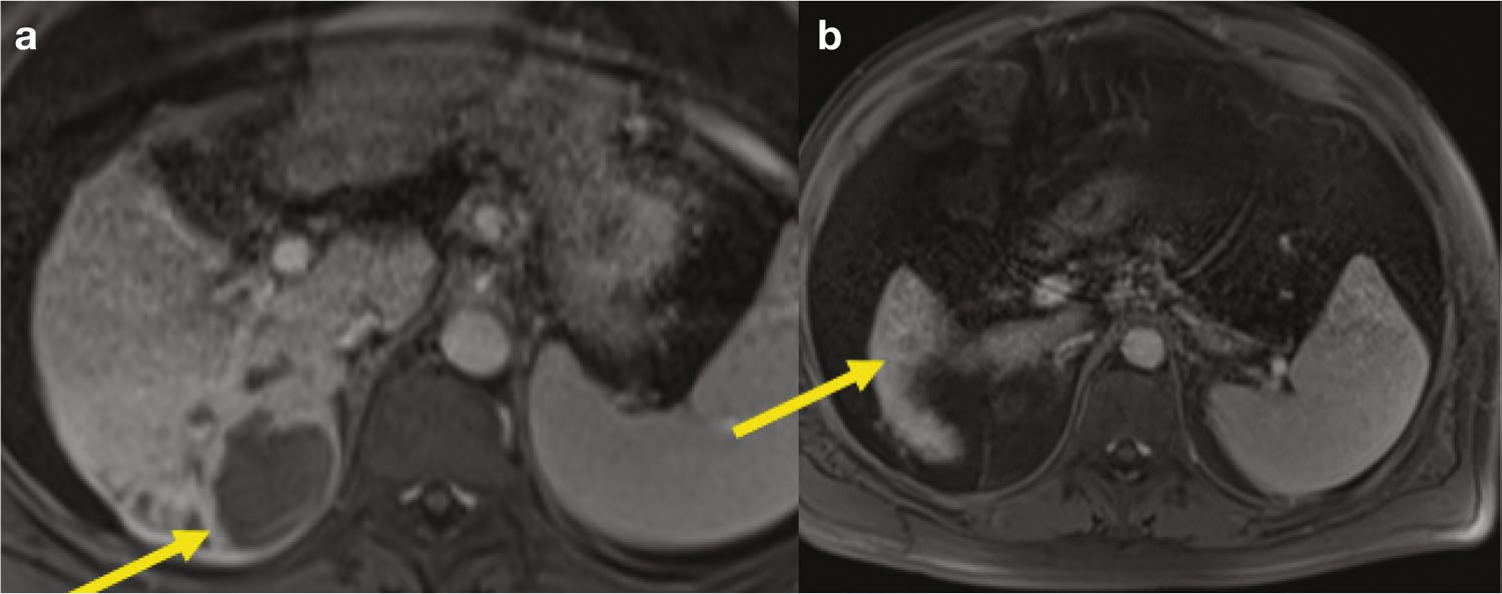
Same patient as in Fig. 3. Gadoxetic acid-enhanced T1-weighted images post-TARE during the PVP at two different levels demonstrate completely necrotic lesion in segment 7 (**a**), and persistently enhancing lesion in segment 6 (arrows) (**b**). *Abbreviations: PVP,* portal venous phase*; TARE,* transarterial radioembolization

The patient has been treated with TARE, and we have an MRI 9 months post treatment showing that the large tumor is completely necrotic and the second (small) tumor is still viable (Fig. 4), so the patient has been successfully downgraded. He was subsequently transplanted with complete tumor necrosis in the segment 7 lesion and persistent viability in the segment 6 lesion.

#### Professor Kudo

This is a very successful case, but unfortunately in Japan, we cannot use TARE. So, maybe we would perform superselective TACE, which could be a good choice here.

#### Professor Vilgrain

The waiting list for liver transplants in certain parts of the United States is long, like in Europe. Does this play a role in deciding whether we should choose TACE versus TARE?

#### Professor Tabrizian

In New York, the waiting list is very long, so we would prefer TARE over TACE. In selected cases, we would treat with TACE, but we have predominantly used TARE. There is a theoretical risk of possible issues of surgical complications during the surgical dissection in patients treated with TARE; however, we have not encountered it in our experience.

#### Dr. Pinato

This is a case that nicely illustrates the importance of multidisciplinary management in HCC. This case illustrates the successful treatment of a Child-Pugh B patient. These patients may present with different grades of dysfunction within Child-Pugh B, for example, without ascites or with ascites. Patients with Child-Pugh B often have a higher risk of portal hypertension and variceal bleeding. Other important factors to consider are demographics and comorbidities.

### Case 3: 69-year-old male with HCV cirrhosis and HCC, status post-SBRT and TARE to the left lobe tumor

#### Professor Vilgrain

**Fig. 5:**
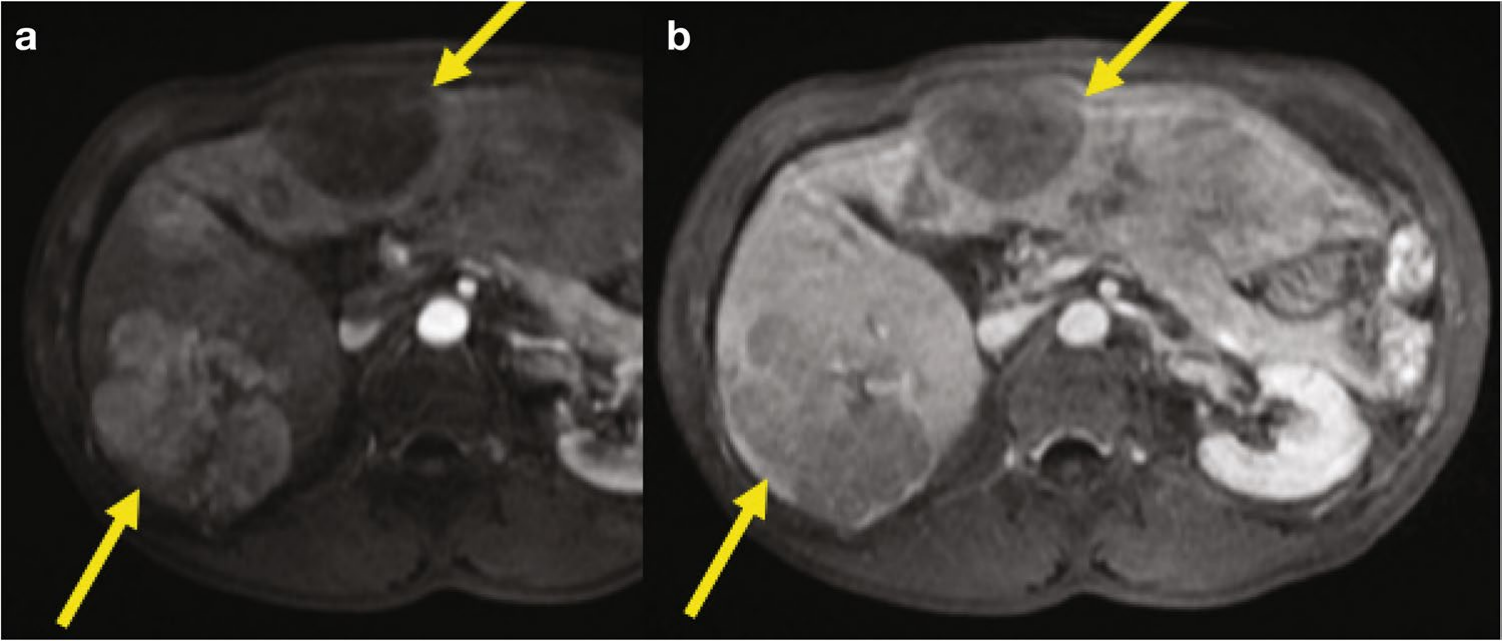
Sixty-nine-year-old male with HCV cirrhosis and HCC. Gadoxetic acid-enhanced T1-weighted images during the arterial (**a**) and PVP (**b**) demonstrate two large infiltrative tumors: an arterially hyperenhancing tumor in the right hepatic lobe (right arrow) and a venous-enhancing tumor in the left hepatic lobe (left arrow). *Abbreviation: PVP,* portal venous phase

This is a patient (Fig. 5) with bilobar disease, who previously received stereotactic body radiotherapy (SBRT) and TARE. Systemic therapy options include immunotherapy (checkpoint inhibitors), TKIs, and the combination of checkpoint inhibitors plus antiangiogenic drugs. Do you think that there is still a role for locoregional therapy?

#### Dr Pinato

This is a patient who has relapsed following locoregional therapy and therefore would benefit from systemic therapy. I do not think that locoregional therapy may help in this case. We obviously have to reflect on all the variables that we discussed before, including performance status, and we have to be mindful of the potential for risk of immunotoxicity and bleeding. Provided this patient is in Child-Pugh A, there is a strong indication for systemic therapy. My preferred choice would be combination immunotherapy (atezolizumab with bevacizumab), with reported response rates as high as 30%, and median overall survival approaching 19 months in first line [56]. TKI therapy, either with sorafenib or lenvatinib as the first line in patients who are not candidates for atezolizumab, would also be a good choice here.

#### Professor Vilgrain

What are the contraindications for the combination of atezolizumab with bevacizumab?

#### Dr Pinato

There is a potential risk of immunotoxicity. We need to assess the underlying chronic liver disease. For example, autoimmune hepatitis would be an exclusion due to the high risk of flare of underlying autoimmune disease. Other types of risk include the risk of bleeding, so we tend to request upper gastrointestinal endoscopy prior to starting treatment. In the IMbrave150 study, patients were allowed to have an endoscopy 6 months prior to starting treatment [26]. If present, varices should be treated either medically or endoscopically. There are no data in patients with a performance status (PS) score > 1, whereas evidence for use in patients with Child-Pugh B is limited to observational studies [57]. So those would be contraindications for this combination therapy.

#### Professor Vilgrain

Professor Kudo: do you share the same experience moving to systemic treatments and maybe a first-line combination?

#### Professor Kudo

This is a Child-Pugh A patient classified as an intermediate stage without extrahepatic spread, so systemic therapy would be the best choice. Among the systemic therapies, atezolizumab plus bevacizumab should be highly considered, with a reported response rate of up to 44% in intermediate-stage HCC [56]. After atezolizumab plus bevacizumab treatment, curative intent therapy can be possible [58].

#### Professor Zhou

I agree with Professor Kudo’s opinion. Systemic therapy should be considered first. If the patient cannot tolerate systemic therapy, TACE can be proposed as another option, at least for the right hepatic lobe tumor, because it is arterially hyperenhancing.

#### Professor Taouli

Can somebody comment on radiation therapy?

#### Dr. Pinato

I think the problem with SBRT is that it has become a fairly orphan type of treatment, due to the fact that the treatment landscape of HCC has moved quickly toward systemic therapy while radiotherapy evolved more slowly and without a specific link to a defined HCC stage [59]. We tend to use SBRT, for example, in cases with very small tumor volumes that are very difficult to access from an interventional radiology point of view, with good response rates. In cases like the one presented here, systemic therapy has proven benefit, so I would be hesitant about utilizing SBRT, due to the fact that it may compromise the opportunity to access highly effective systemic therapy in patients with aggressive tumor biology.

#### Professor Ringe

I agree with Dr. Pinato. It is a large tumor and we also favor SBRT in smaller lesions that cannot be easily accessed by the interventional radiologist. In this particular case, the patient had already had radiation therapy and TARE, so there is an increased risk of radiation-induced liver disease, which may compromise further treatment.

#### Professor Vilgrain

This is a very good point.

#### Professor Tabrizian

We have had select cases with large tumors and portal vein invasion where we did a combination of SBRT and TARE or TACE with good outcomes in terms of controlling the portal vein thrombosis.

#### Professor Vilgrain

**Fig. 6:**
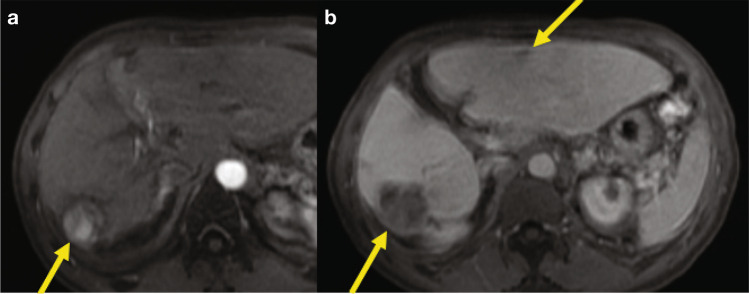
Follow-up post nivolumab therapy demonstrates partial response. Gadoxetic acid-enhanced T1-weighted images during the arterial (**a**) and PVP (**b**) demonstrate partial response with decreased size of the arterially hyperenhancing tumor in right hepatic lobe (right arrow) and near-complete resolution of left hepatic lobe tumor (left arrow). *Abbreviation: PVP*, portal venous phase

Based on our discussion, we can conclude that SBRT is more debated than other treatments. There are certainly cases where SBRT works well, and again these treatments should be discussed during multidisciplinary team sessions. This case also illustrates our initial experience with immune checkpoint inhibitors (such as nivolumab) in patients with advanced/unresectable HCC. In this patient, follow-up post nivolumab therapy demonstrated partial response (Fig. 6).

## Summary

The 10th Global Forum provided an opportunity for multidisciplinary specialists to discuss their approaches to the management of early-, mid-, and late-stage HCC, in the form of presentations and by discussion of challenging case studies.

## Abbreviations

AASLD: American Association for the Study of Liver Diseases
AFP: Alfa-fetoprotein
ASCO: American Society of Clinical Oncology
BCLC: Barcelona Clinic Liver Cancer
CECT: Contrast-enhanced computed tomography
EASL: European Association for the Study of the Liver
ECOG PS: Eastern Cooperative Oncology Group Performance Status
ESMO: European Society for Medical Oncology
FLR: Functional liver reserve
MWA: Microwave ablation
PD-1: Programmed cell death protein 1
PD-L1: Programmed death ligand 1
PVP: Portal venous phase
RFA: Radiofrequency ablation
SBRT: Stereotactic body radiotherapy
SORAMIC: Sorafenib and Micro-therapy Guided by Primovist Enhanced MRI in Patients with Inoperable Liver Cancer (trial)
STORM: Sorafenib as Adjuvant Treatment in the Prevention of Recurrence of Hepatocellular Carcinoma
TARE: Transarterial radioembolization
TKI: Tyrosine kinase inhibitor
VEGF: Vascular endothelial growth factor

## Declarations

### Guarantor

The scientific guarantor of this publication is Bachir Taouli, MD, MHA.

### Statistics and biometry

No complex statistical methods were necessary for this paper.

### Study subjects or cohorts overlap

Not applicable as this is a review paper.

### Methodology

- Not applicable as this is a review paper
