# Correction: Consensus report from the 10th global forum for liver magnetic resonance imaging: multidisciplinary team discussion

**DOI:** 10.1007/s00330-023-10342-7

**Published:** 2023-11-06

**Authors:** Bachir Taouli, Ahmed Ba-Ssalamah, Julius Chapiro, Jagpreet Chhatwal, Kathryn Fowler, Tae Wook Kang, Gesine Knobloch, Dow-Mu Koh, Masatoshi Kudo, Jeong Min Lee, Takamichi Murakami, David J. Pinato, Kristina I. Ringe, Bin Song, Parissa Tabrizian, Jin Wang, Jeong Hee Yoon, Mengsu Zeng, Jian Zhou, Valérie Vilgrain

**Affiliations:** 1 Department of Diagnostic, Molecular, and Interventional Radiology, BioMedical Engineering and Imaging Institute, Icahn School of Medicine at Mount Sinai, New York, NY USA; 2 Department of Biomedical Imaging and Image-Guided Therapy, Medical University of Vienna, Vienna, Austria; 3 Department of Radiology and Biomedical Imaging, Yale School of Medicine, New Haven, CT USA; 4 Department of Radiology, Institute for Technology Assessment, Massachusetts General Hospital, Harvard Medical School, Boston, MA USA; 5 Department of Radiology, University of California San Diego, La Jolla, CA USA; 6 Department of Radiology and Center for Imaging Science, Samsung Medical Center, Sungkyunkwan University School of Medicine, Seoul, South Korea; 7 Global Medical and Clinical Affairs and Digital Development, Radiology, Bayer Pharmaceuticals, Berlin, Germany; 8 Department of Diagnostic Radiology, Royal Marsden Hospital, Sutton, UK; 9 Department of Gastroenterology and Hepatology, Kindai University Faculty of Medicine, Osaka, Japan; 10 Department of Radiology, Seoul National University Hospital and Seoul National University College of Medicine, Seoul, South Korea; 11 Department of Radiology, Kobe University Graduate School of Medicine, Kobe, Japan; 12 Department of Surgery & Cancer, Imperial College London, Hammersmith Hospital, London, UK; Division of Oncology, Department of Translational Medicine, University of Piemonte Orientale, Novara, Italy; 13 Department of Diagnostic and Interventional Radiology, Hannover Medical School, Hannover, Germany; 14 Department of Radiology, West China Hospital, Sichuan University, Chengdu, People’s Republic of China; 15 Recanati/Miller Transplantation Institute, Icahn School of Medicine at Mount Sinai, New York, NY USA; 16 Department of Radiology, Third Affiliated Hospital of Sun Yat-Sen University, Guangzhou; Liver Disease Hospital, Sun Yat-Sen University, Guangzhou, People’s Republic of China; 17 Department of Radiology, Zhongshan Hospital, Fudan University, Shanghai, People’s Republic of China; 18 Liver Cancer Institute, Zhongshan Hospital, Fudan University, Shanghai, People’s Republic of China; 19 Université Paris Cité and Department of Radiology, Assistance-Publique Hôpitaux de Paris, APHP Nord, Hôpital Beaujon, Clichy, France

**Correction: European Radiology**

**https://doi.org/10.1007/s00330-023-09919-z**

The article “Consensus report from the 10th global forum for liver magnetic resonance imaging: multidisciplinary team discussion,” written by Bachir Taouli, Ahmed Ba-Ssalamah, Julius Chapiro, Jagpreet Chhatwal, Kathryn Fowler, Tae Wook Kang, Gesine Knobloch, Dow-Mu Koh, Masatoshi Kudo, Jeong Min Lee, Takamichi Murakami, David J. Pinato, Kristina I. Ringe, Bin Song, Parissa Tabrizian, Jin Wang, Jeong Hee Yoon, Mengsu Zeng, Jian Zhou, and Valérie Vilgrain, was originally published electronically on the publisher’s internet portal on 13 July 2023, without open access. With the author(s)’ decision to opt for Open Choice, the copyright of the article changed on 28 September 2023 to © The Author(s) 2023 and the article is forthwith distributed under a Creative Commons Attribution 4.0 International License, which permits use, sharing, adaptation, distribution and reproduction in any medium or format, as long as you give appropriate credit to the original author(s) and the source, provide a link to the Creative Commons licence, and indicate if changes were made. The images or other third party material in this article are included in the article’s Creative Commons licence, unless indicated otherwise in a credit line to the material. If material is not included in the article’s Creative Commons licence and your intended use is not permitted by statutory regulation or exceeds the permitted use, you will need to obtain permission directly from the copyright holder. To view a copy of this licence, visit http://creativecommons.org/licenses/by/4.0/.

The original article has been corrected.

